# Chemically different non-thermal plasmas target distinct cell death pathways

**DOI:** 10.1038/s41598-017-00689-5

**Published:** 2017-04-04

**Authors:** Oleg Lunov, Vitalii Zablotskii, Olexander Churpita, Mariia Lunova, Milan Jirsa, Alexandr Dejneka, Šárka Kubinová

**Affiliations:** 1 Institute of Physics of the Academy of Sciences of the Czech Republic, Prague, 18221 Czech Republic; 2 Institute for Clinical & Experimental Medicine (IKEM), Prague, 14021 Czech Republic; 3 Institute of Experimental Medicine AS CR, Prague, 14220 Czech Republic

## Abstract

A rigorous biochemical analysis of interactions between non-thermal plasmas (NTPs) and living cells has become an important research topic, due to recent developments in biomedical applications of non-thermal plasmas. Here, we decouple distinct cell death pathways targeted by chemically different NTPs. We show that helium NTP cells treatment, results in necrosome formation and necroptosis execution, whereas air NTP leads to mTOR activation and autophagy inhibition, that induces mTOR-related necrosis. On the contrary, ozone (abundant component of air NTP) treatment alone, exhibited the highest levels of reactive oxygen species production leading to CypD-related necrosis via the mitochondrial permeability transition. Our findings offer a novel insight into plasma-induced cellular responses, and reveal distinct cell death pathways triggered by NTPs.

## Introduction

Over the last decade, biochemical interactions between non-thermal plasmas (NTPs) and living objects have steadily gained increased attention due to recent developments in plasma medicine^1^. NTP demonstrated numerous biological and medical applications, ranging from microorganism deactivation and wound healing, to cancer treatment^1, 2^. However, there is still a lack of knowledge on cellular targets as well as on the biochemical mechanisms of plasma action.

NTPs represent partially ionized gases, that contain a chemically complex and reactive environment. Typical NTP composition is a mixture consisting of ions, electrons, free radicals, UV radiation, and neutral molecules. Nowadays, it is relatively easy to manipulate plasma chemical composition utilizing different gases, such as helium (He), argon (Ar), nitrogen (N_2_), ambient air or a mixture of gases^1, 3^. Consequently, the extent of biological responses to NTPs can vary enormously depending on the physical and chemical characteristics of plasma, the experimental and ambient conditions and the biological target^4, 5^. Generally, it is believed that the principal mode of plasma-cell interaction is due to the accumulation of reactive oxygen (ROS) and reactive nitrogen species (RNS) that can be generated in or transferred into, the liquid phase surrounding the biological target^6, 7^. Numerous studies have shown an ability of non-thermal plasma to generate many kinds of reactive species: O, ^•^OH, O_2_^•−^, ^1^O_2_, NO^•^, NO_2_^•^, H_2_O_2_, NO_2_^−^, NO_3_^−^, O_3_, for review see ref. 8. However, the exact composition and concentration range of these ROS and RNS in the plasma-treated liquids may grossly vary, depending on the carrier gas that forms the NTP^9^.

In general, previous research on plasma-cell interactions has been focused on NTPs produced by utilizing one particular carrier gas. Hence, it is of great importance to compare and discriminate the biological effects triggered by plasma originating from the discharges produced in different carrier gases. Furthermore, the information concerning the comparison of the biological effects of non-thermal plasma and ozone (an abundant composite of NTP) is basically lacking. So far, the exact molecular mechanisms of how NTP changes cellular functionality are not known. It is believed, that the main biological consequences of cell treatment with NTP are intracellular RNS and ROS appearance^10–12^. However, in literature, there is substantial disagreement on the molecular consequences of NTP cell treatment^13, 14^. On one hand, it has been shown that NTP treatment results in an apoptosis triggering^7, 13, 15^. Other groups show that NTP induces a predominantly necrotic cell death^14, 16^. Given that there is a substantial molecular crosstalk between apoptosis and necrosis pathways^17, 18^ and there is a large variability in the design and construction of plasma producing sources^8, 19^, it is no surprise that biological effects triggered by NTP differ so much. Moreover, studies that compare the effects of chemically distinct plasmas generated from the same plasma source have not been thoroughly performed. Therefore, the aim of this study is to investigate and compare the effects of two chemically different NTPs and ozone, on physiological and pathophysiological cellular functions. Bearing in mind the discrepancies in literature concerning cell death pathways triggered by NTP, we hypothesized that the chemical composition of NTP would significantly affect which signaling pathway will be activated by distinct NTPs. This study gathers information on the potential molecular targets of different non-thermal plasmas and provides tentative molecular mechanisms of NTPs action on living cells.

## Results

### Non-thermal plasma characterization

**Figure 1:**
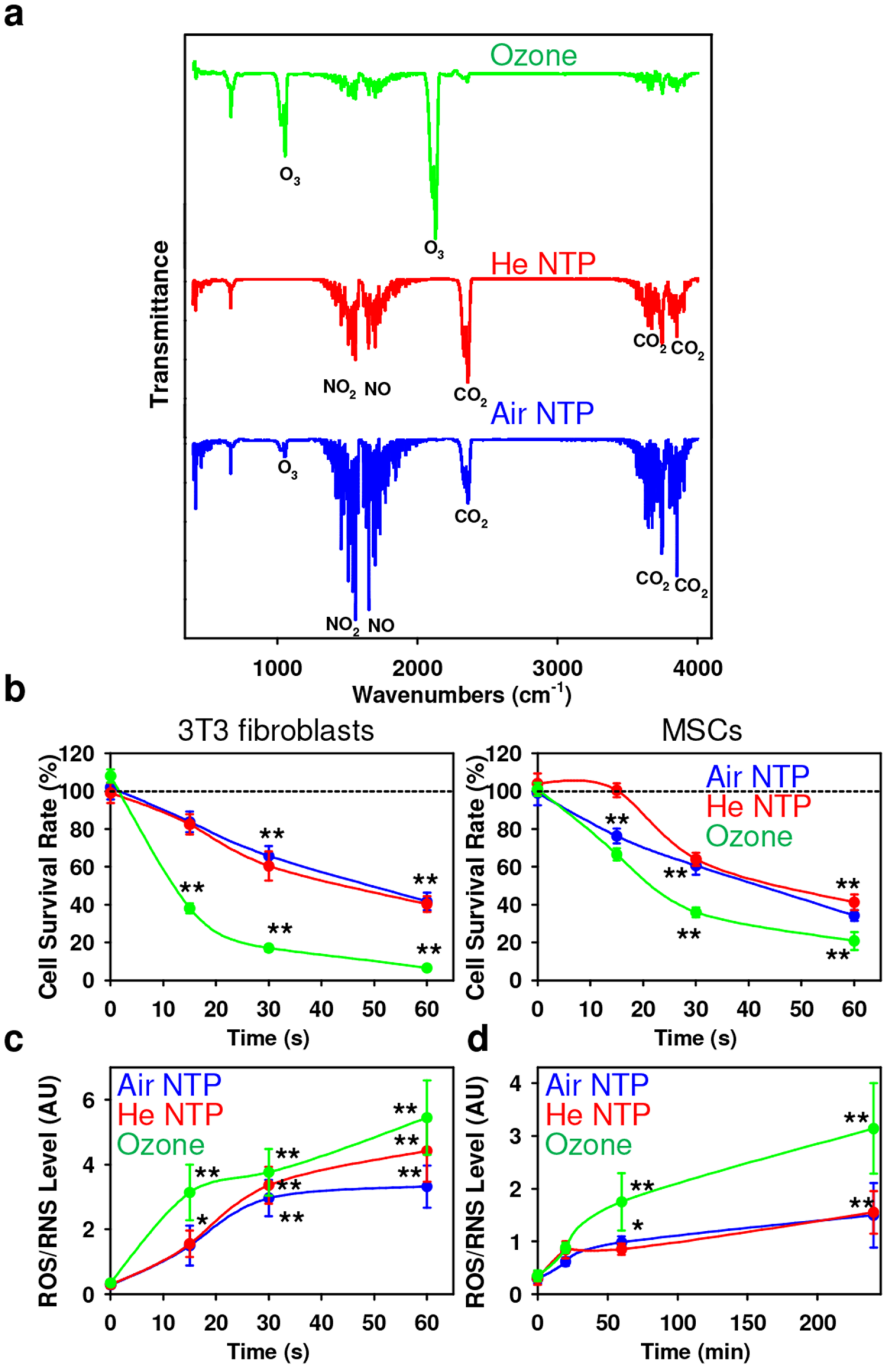
Physicochemical characterization of the chemically distinct non-thermal plasmasand cytotoxic effects elicited by different plasmas. (**a**) The fourier transform infrared spectroscopy (FT-IR) of air, helium NTPs and ozone. (**b**) Cell viability as detected by the WST-1 assay of 3T3 fibroblasts and MSCs treated with air, helium NTPs or ozone for indicated time periods, measured 24 h after exposure. The data were normalized to control values (no exposure), which were set as 100% cell viability. Readings were done in quadruplicates, data are present as mean ± SD, n = 3 (three independent experiments). One-way ANOVA with Newman–Keuls multiple comparison test was used; t = 0 time point serving as control, *P < 0.05 **P < 0.01. (**c**) Dose-dependent and (**d**) time-dependent ROS/RNS induction by air, helium NTPs and ozone. Cells were exposed to air, helium NTPs or ozone, followed by ROS measuring, using the cellular ROS/RNS detection kit (Abcam) by spectrofluorometry. Readings were done in quadruplicates, data are present as mean ± SEM, n = 3 (three independent experiments). One-way ANOVA with Newman–Keuls multiple comparison test was used; t = 0 time point serving as control, *P < 0.05 **P < 0.01.

In order to answer such important questions, we carefully examined and compared the effects of two types of non-thermal plasmas (air and helium) and ozone, exerted on living cells. We utilized a previously characterized plasma system^5, 20^ working at atmospheric pressure to study plasma-cell interactions (see Fig. S1 in Supporting Information). Fourier transform infrared (FTIR) transmittance spectra gave an overview of the composition of the air and helium (He) NTPs analyzed in the jet (Fig. 1a). Use of transmittance/absorption spectroscopy is more preferable for plasma composition analysis than commonly used emission spectroscopy. It allowed us to obtain complementary information to plasma characterization given in ref. 5. Detailed spectral and compositional characteristics of the air and He NTPs have been published previously^5, 21^. Importantly, to produce either air or He NTPs we utilized the same plasma system. It is known that high doses of NTP can change media pH^22^. However, short-term exposure, less than 4 min, does not lead to pH changes in media^9, 23, 24^. Indeed, in our case, the pH did not change after 1 min of either air or helium plasma irradiation of cell culture media. It is worth noting, that the UV radiation generated by the plasma source could be responsible for the activation of cellular processes. However, power measurements of UV production by plasma system showed that the power density of the emitted UV radiation for air was lower than 1 μW/cm^2^ and for He NTP– 3 ± 1 μW/cm^2^, which is at least one order of magnitude lower than the minimal power density needed to have any effect on living cells^25–27^. Additionally, according to the SCCP European Commission Report 0949/05, the maximum allowed UV dose rate is 50 μW/cm^2^, which is one order of magnitude higher than in our case^27^. Moreover, we^28^ and others^29, 30^ have previously shown that the UV radiation is not the dominant biological agent of non-thermal plasmas of such ion density and energy. Detailed NTP treatment procedure and plasma system characterization is given in the Materials and Methods section. Taken together, composition of air NTP was substantially different in comparison with He NTP and ozone generated by a conventional ozone generator. Importantly, air NTP produced elevated levels of NO, O_3_ and ${{\rm{N}}}_{2}^{+}$ in comparison with He NPT. Contrarily, in He NTP a substantial amount of He ions were detected, which were lacking in air NTP^5, 21^.

### Cytotoxic effects induced by different plasmas via ROS/RNS accumulation

Ourselves and others have repeatedly shown the acute cytotoxicity of distinct NTPs on different cell lines^4–7, 14, 31^. However, a comparative study of the cytotoxic effects of different NTPs (generated using the same plasma reactor) on the same cellular model system has not been performed. Furthermore, numerous studies possess ROS/RNS generation by NTPs as the main effector on mammalian cell functionality^4–7, 14, 31^. Thus, NTP-induced biological effects span from increased proliferation^6^ to cell death, through the formation of intracellular ROS^4–7, 14, 31^. According to Fig. 1b, cytotoxic effects induced by both NTPs and ozone were dose-dependent. There was no significant difference between air and He NTPs at higher doses (namely 30 and 60 s) in exerting cytotoxicity toward the test cell lines (Fig. 1b): 3T3 fibroblasts and mesenchymal stem cells (MSCs). It is worth noting, that the air NTP showed significantly higher toxicity at a 15 s dose in comparison with He NTP (Fig. 1b). However, ozone appeared to induce significantly higher instances of cell death (Fig. 1b).

Taking into account the general paradigm that primarily ROS/RNS are responsible for NTPs-induced cellular effects^6–8^, we, therefore, were interested in the generation of intracellular ROS/RNS followed by plasma treatment. Consistently with previously published results, we found that both NTPs and ozone triggered a time- and dose-dependent ROS production in both cell lines (Fig. 1c,d). It is clearly shown (Fig. 1c), that both NTPs and ozone induced dose-dependent ROS accumulation in cells with the highest amount of ROS, produced after long term (60 s exposure) plasma irradiation (Fig. 1c). It is worth noting, that both short-lived, with a half-life of μs range (O, ^•^OH, O_2_^•−^, ^1^O_2_, NO^•^, NO_2_^•^), and relatively long-lived with a half-life of ms range (H_2_O_2_, NO_2_^−^, NO_3_^−^, O_3_) species, have been detected in the NTPs and also in the plasma-treated liquids^32, 33^. At this point, the question arises on how such a short-lived ROS could potentially penetrate through the medium all the way to the cells. However, it has been shown that long-lived O_3_-generated reactive oxygen intermediates (ROIs) are formed, and the chemical lifetime of these intermediates exceeds 100 seconds^34^. Furthermore, it has been shown that plasma can deliver ROS up to 1.5 mm below the tissue surface^32, 35^. Another important component of NTP jet, ions, has a penetration depth ranging from tenth μm to a few mm^36, 37^. We next monitored the generation of intracellular ROS following plasma treatment (Fig. 1d). Following plasma treatment, intracellular ROS levels increased after 1 or 4 h post NTP treatment, but not after 30 min post treatment (Fig. 1d). These data suggest that plasma treatment induces the generation and accumulation of intracellular ROS. Interestingly, ozone induced a significantly higher production of total intracellular ROS/RNS than air and He NTPs (Fig. 1c,d). Treatment with *N-acetyl-L-cysteine* (NAC, a powerful free radical scavenger) completely abolished the cytotoxic effects of air and He NTPs and ozone, confirming the role of ROS in the induction of cell death (see Fig. S2 in Supporting Information).

### Oxidative stress induced by exposure to plasma damages DNA, but does not result in apoptosis execution

**Figure 2:**
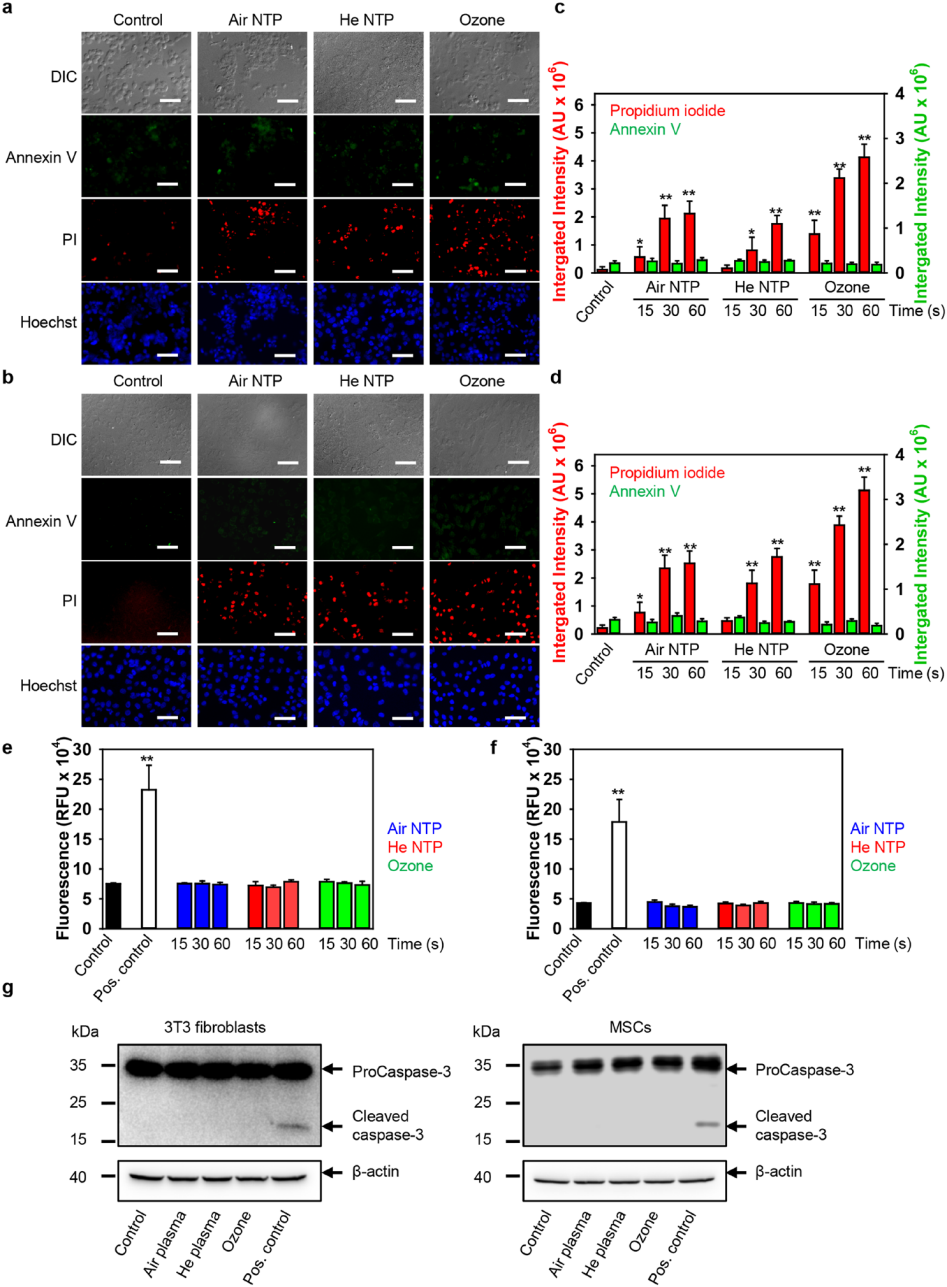
Analysis of apoptosis hallmarks after plasma treatment. 3T3 fibroblasts (**a**) or MSCs (**b**) were treated with air, helium NTPs or ozone for 60 s, then 4 h after treatment cells were labeled with Hoechst nuclear stain – blue dye, annexin V – green dye and propidium iodide – red dye. Labelled cells were imaged with fluorescence microscopy. Representative images out of three independent experiments are shown. Scale bar 100 µm. (**c**) Annexin V and PI image quantification of plasma-treated 3T3 fibroblasts. Annexin V – green dye and propidium iodide – red dye fluorescence intensities were analyzed with ImageJ. The data present mean values of three independent experiments. In each experiment 5 randomly selected fields for each sample were quantified. Data are expressed as means ± SEM (n = 3), *P < 0.05 **P < 0.01. (**d**) Annexin V and PI image quantification of plasma-treated MSCs. Annexin V – green dye and propidium iodide – red dye fluorescence intensities were analyzed with ImageJ. The data present the mean values of three independent experiments. In each experiment 5 randomly selected fields for each sample were quantified. Data are expressed as means ± SEM (n = 3), *P < 0.05 **P < 0.01. Caspase-3 activation assay in 3T3 fibroblasts (**e**) and MSCs (**f**). Cells were stimulated with air, helium NTPs and ozone for the indicated period of time. Further, 4 h post-treatment cells were incubated with caspase-3 inhibitor VAD-FMK conjugated to FITC (FITC-VAD-FMK). Following staining, cells were analyzed using a fluorescent microplate reader (Tecan Infinite® 200 PRO). Readings were done in quadruplicates. As a positive control, cells were treated with 2 μM staurosporine for 3 h. The data present the mean values of four independent experiments. Data are expressed as means ± SEM (n = 4). (**g**) Air, helium NTPs and ozone do not induce caspase-3 activation. 3T3 fibroblasts and MSCs were stimulated with air, helium NTPs and ozone for 30 s. Cells were analyzed by Western immunoblotting 4 h post-treatment. Actin – control of equal protein loading. Representative blots out of three independent experiments are shown. As a positive control, cells were treated with 2 μM staurosporine for 3 h.

Further, to clarify if the time dependent increase of intracellular ROS induces apoptosis and if reduction in cell viability can be explained by apoptosis, annexin V-propidium iodide staining was performed after NTP treatment. Indeed, annexin V-propidium iodide (PI) double staining suggested that both NTPs and ozone induce either late stage apoptotic or necrotic cell death (Fig. 2a–d). Additionally, to confirm that NTP does not induce apoptosis, we performed caspase-3 activity assay (Fig. 2e,f). Caspase 3 is an executioner of apoptosis, and its activation constitutes irreversible morphological changes characteristic of the apoptotic process, such as DNA degradation, chromatin condensation, and membrane blebbing^38^. Fluorometric analysis of caspase 3 activation in cells treated with either two NTPs or ozone, showed that air, He NTPs and ozone did not induce apoptotic cell death in 3T3 fibroblasts and MSCs (Fig. 2e,f). Consistent with the fluorometric analysis, the caspase 3 activation after NTPs and ozone treatments was not detected utilizing immunoblot analysis (Fig. 2g). It is worth noting here that treatment with staurosporine (a well-known apoptosis inducing compound) of both cell lines resulted in caspase 3 activation (Fig. 2e–g).

**Figure 3:**
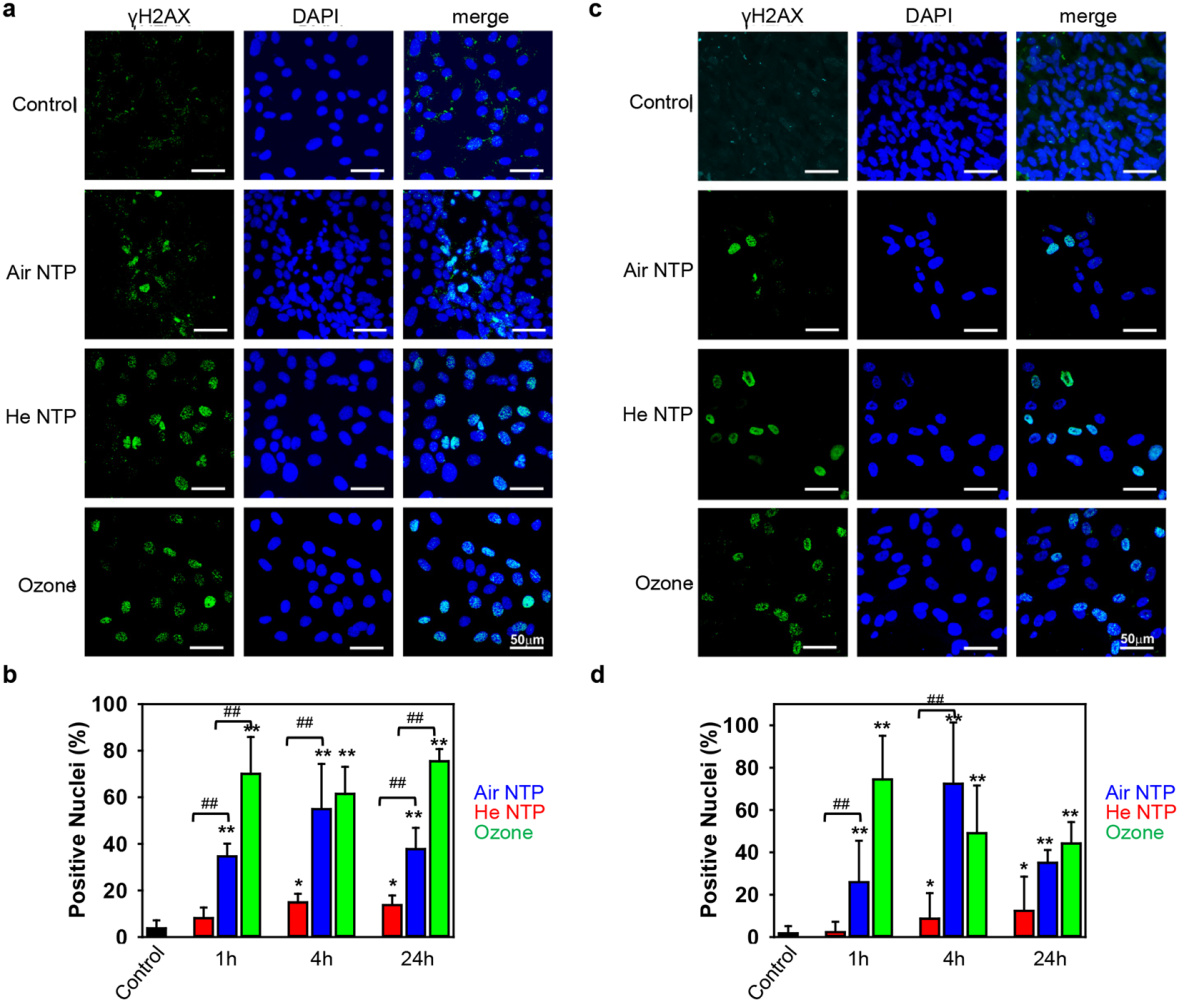
Induction of DNA damage by different NTPs and ozone. (**a**) 3T3 fibroblasts and (**c**) MSCs were treated with air, helium NTPs or ozone for 30 s, then 4 h after treatment cells were assessed by immunofluorescence utilizing an antibody to γ-H2AX (green) and nuclei (blue). (**b**) 3T3 fibroblasts and (**d**) MSCs treated with air, helium NTPs or ozone for 30 s. Cell were stained for nuclei (blue), and phosphorylated form of γ-H2AX (green) 1, 4 and 24 h post-treatment. Labeled cells were then imaged using epi-fluorescent microscopy. Quantitative analysis was carried out by counting the number of immunoreactive cells as the percentage of the total number of viable cells, as determined by DAPI staining. Image was processed and quantified with ImageJ software (NIH, Bethesda, MD, USA). Scale bar 50 µm. *P < 0.05 **P < 0.01 versus controls, ^##^ *P* < 0.01, mean ± SEM, n = 3.

These data are in line with our, and others, previously published results^5, 14, 39^. However, other studies show apoptosis triggered by NTPs via the formation of intracellular ROS^6, 7^. These discrepancies prompted us to investigate in detail what biochemical pathways might be involved in NTP-induced cell death. Indeed, ROS accumulation resulted in DNA damage, revealed by phosphorylation of H2AX (Fig. 3a–d), a histone variant that is phosphorylated in response to DNA damage, and associated with oxidative stress^40^. Consistent with cytotoxicity, ozone induced the highest DNA damage. It is worth noting here, that air NTP treatment resulted in higher DNA damage in comparison with He NTP (Fig. 3a–d). Taken together, these data clearly show that plasma treatment, despite induction DNA oxidative damage, does not trigger apoptosis. Thus, we searched for other possible mechanisms of cell death.

### Helium non-thermal plasma triggers necroptosis

**Figure 4:**
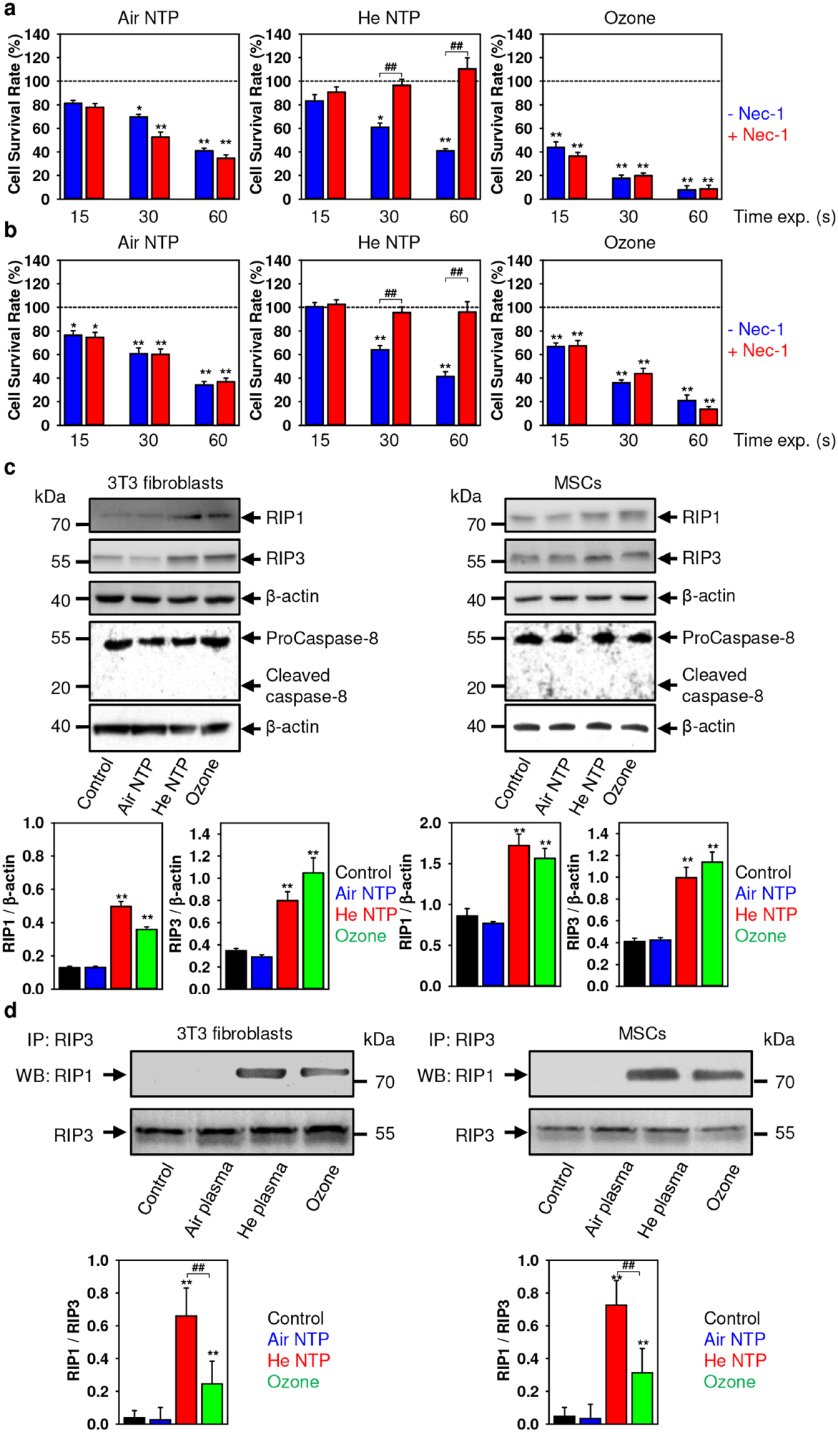
Necrostatin-1 (Nec-1, a potent and selective inhibitor of necroptosis) antagonizes the He NTP-induced cytotoxicity. Cell viability as detected by the WST-1 assay of (**a**) 3T3 fibroblasts and (**b**) MSCs treated with air, helium NTPs or ozone for indicated time periods with supplementation of 10 µM Nec-1, measured 24 h after exposure. Readings were done in quadruplicates. The data present the mean values of four independent experiments. Data are expressed as means ± SEM (n = 4), *P < 0.05 **P < 0.01. (**c**) He NTP and ozone treatment induces RIP1 and RIP3 upregulation (full blots of RIP1 and RIP3 are presented in Fig. S5 in Supporting Information) without concomitant activation of caspase-8. 3T3 fibroblasts and MSCs were treated with air, helium NTPs or ozone for 30 s. Cells were analyzed by Western immunoblotting 4 h after treatment. Actin – control of equal protein loading. The graphs show densitometric quantification of RIP1 and RIP3 Western immunoblots, *P < 0.05 **P < 0.01, mean ± SEM, n = 3. Representative blots out of three independent experiments are shown. (**d**) RIP3 complexes isolated by immunoprecipitation from either 3T3 fibroblasts or MSCs treated with air, He NTPs and ozone for 30 s. The graph shows densitometric quantification of the respective immunoblots, *P < 0.05 **P < 0.01, mean ± SEM, n = 3. Representative blots out of three independent experiments are shown.

Of note, ROS/RNS have been implicated in biochemically distinct death pathways, e.g. apoptosis, autophagy, necrosis, necroptosis^41–43^. Furthermore, there is a signaling crosstalk between these cascades of death pathways via ROS^18, 43^. Importantly, a recent study highlighted that NTP may induce a combination of autophagy, apoptosis or necrosis^16^. Indeed, cytotoxicity studies with necrostatin-1 (Nec-1, a well-known inhibitor of necroptosis^44^) revealed, that only He NTP-treated cells were protected by Nec-1 supplementation (Fig. 4a,b). Taken together, these data suggest that He NTP presumably induces necroptosis, and on the contrary, air NTP and ozone trigger other types of necrotic cell death.

Nowadays it is widely accepted, that necroptosis is mediated by a signaling complex called necrosome, which consists of the following major components: receptor-interacting protein RIP1, RIP3, and mixed-lineage kinase domain-like (MLKL)^45^. As we expected, He NTP treatment led to RIP1, RIP3 upregulation in both cell lines (Fig. 4c).

These results nicely correlate with cytotoxicity inhibition by Nec-1 (Fig. 4a,b). Importantly, there was no caspase-8 activation (a well-known inhibitor of necrosome activity^42, 45^) (Fig. 4c). However, we found unexpectedly RIP1, RIP3 upregulation in both cell lines after ozone treatment (Fig. 4c). It is worth noting that murine 3T3 fibroblast and human MSC cell cultures are commonly used as a model of wound healing in the non-thermal plasma field of research^5, 8^. Therefore, we utilized these two commonly accepted cell cultures as model systems for our experiments using non-thermal plasma. Additionally, our intention was to attract the plasma medicine society to careful selection of the cell culture model, as far as there is growing awareness of the limitations of some widely used murine disease models^46, 47^. Furthermore, to prove necroptosis triggering induced by He NTP, we performed a co-immunoprecipitation assay of RIP3-RIP1 complexes (Fig. 4d). Immunoprecipitation with anti-RIP3 antibody showed that either He NTP or ozone but not air NTP treatment resulted in a complex formation between RIP3 and RIP1, which is required for necroptosis triggering^48^. Importantly, the ratio between RIP3 and RIP1 in complexes formed after He NTP treatment was significantly higher in comparison with complexes formed after ozone treatment (Fig. 4d). Recently, it was shown that the ratio between RIP3 and RIP1 proteins in necrosome complexes determines the activity of the necrosome^49^. These results led us to a tentative conclusion that ozone treatment leads to a non-active necrosome complex formation. Further, we looked at what could be the reason for the non-active necrosome complex formation upon ozone treatment.

**Figure 5:**
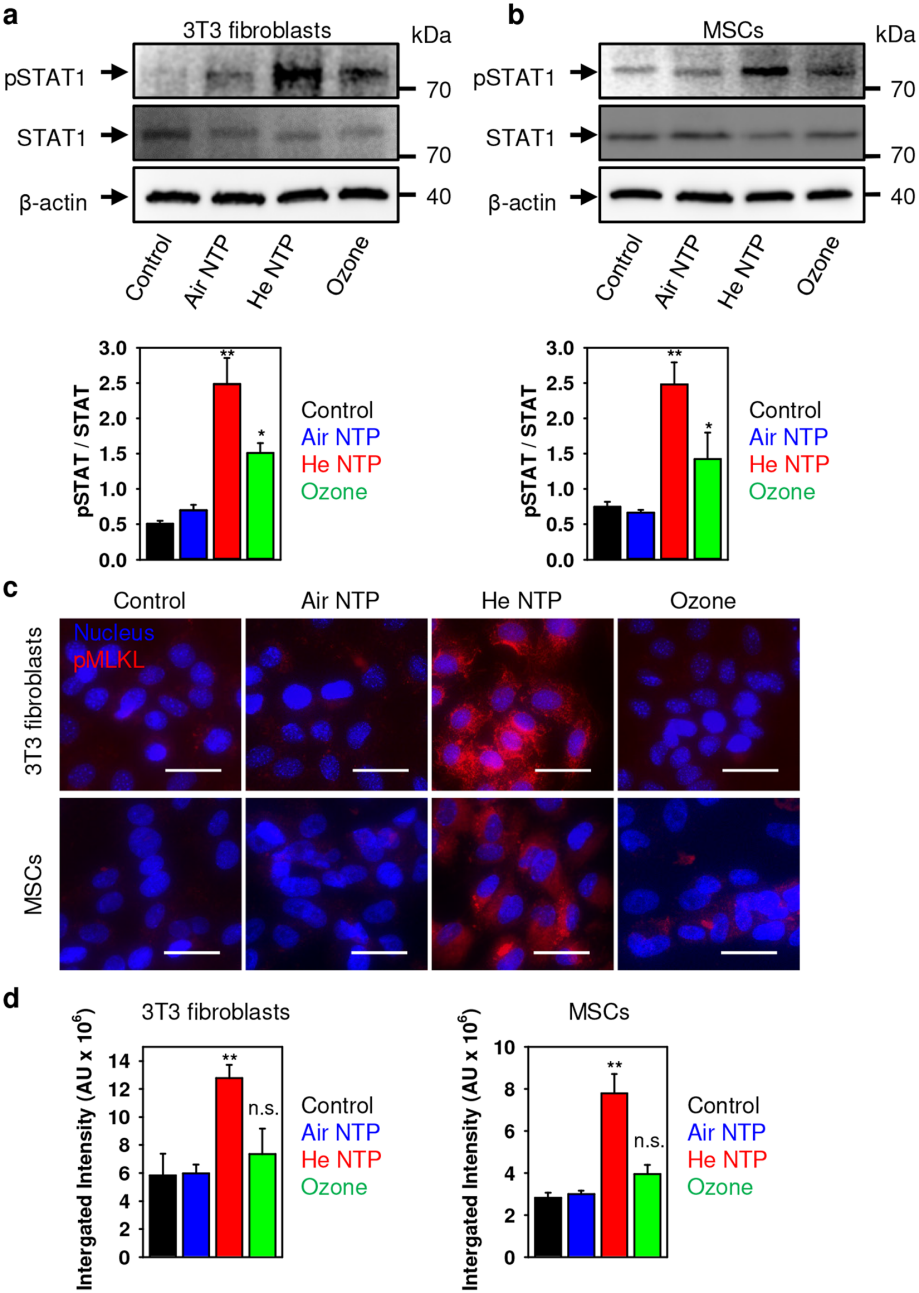
He NTP and ozone treatment induces STAT1 phosphorylation. (**a**) 3T3 fibroblasts and (**b**) MSCs were treated with air, helium NTPs or ozone for 30 s. Cells were analyzed by Western immunoblotting 4 h after treatment. Actin – control of equal protein loading. The graphs show densitometric quantification of the respective Western immunoblots, *P < 0.05 **P < 0.01, mean ± SEM, n = 3. Representative blots out of three independent experiments are shown. (**c**) Effects of air, helium NTPs and ozone on MLKL activation. Representative pictures of 3T3 fibroblasts and MSCs treated with air, helium NTPs or ozone for 30 s. Cell were stained for nuclei (blue), and the phosphorylated form of MLKL (red). Labeled cells were then imaged using epi-fluorescent microscopy, and the image was processed with ImageJ software (NIH, Bethesda, MD, USA). Scale bar 50 µm. (**d**) pMLKL image quantification of plasma-treated 3T3 fibroblasts and MSCs, labeled as described in (**c**). pMLKL fluorescence intensities were analyzed with ImageJ. The data present mean values of three independent experiments. In each experiment 5 randomly selected fields for each sample were quantified. Data are expressed as means ± SEM (n = 3), *P < 0.05 **P < 0.01.

It has been shown that STAT1 phosphorylation coincides with the activation of Rip3 kinase and supports necroptosis^50^. Therefore, we hypothesized that under ozone treatment non-active necrosome complex is formed due to the lack of STAT1 support. However, both ozone and He NTP treatment resulted in STAT1 phosphorylation in both cell lines (Fig. 5a,b). In addition, to prove necroptosis induction by He NTP and non-active necrosome formation under ozone treatment, we investigated MLKL phosphorylation. MLKL phosphorylation has been implicated in necroptosis execution^51^. Immunofluorescent staining (Fig. 5c) and its quantification (Fig. 5d) clearly showed that only after He NTP treatment, a significantly upregulated phosphorylated form of MLKL was detected in both cell lines. Additionally, western blot analysis(see Fig. S3 in Supporting Information) confirmed MLKL phosphorylation only after He NTP treatment, confirming our hypothesis that He NTP induces necroptosis while ozone treatment results in other necrotic cells death. Further, we pursued questions on what type of death induces air NTP and ozone, and what supports necroptosis execution after He NTP treatment.

### Air non-thermal plasma and ozone treatment results in activation of distinct necrotic pathways

**Figure 6:**
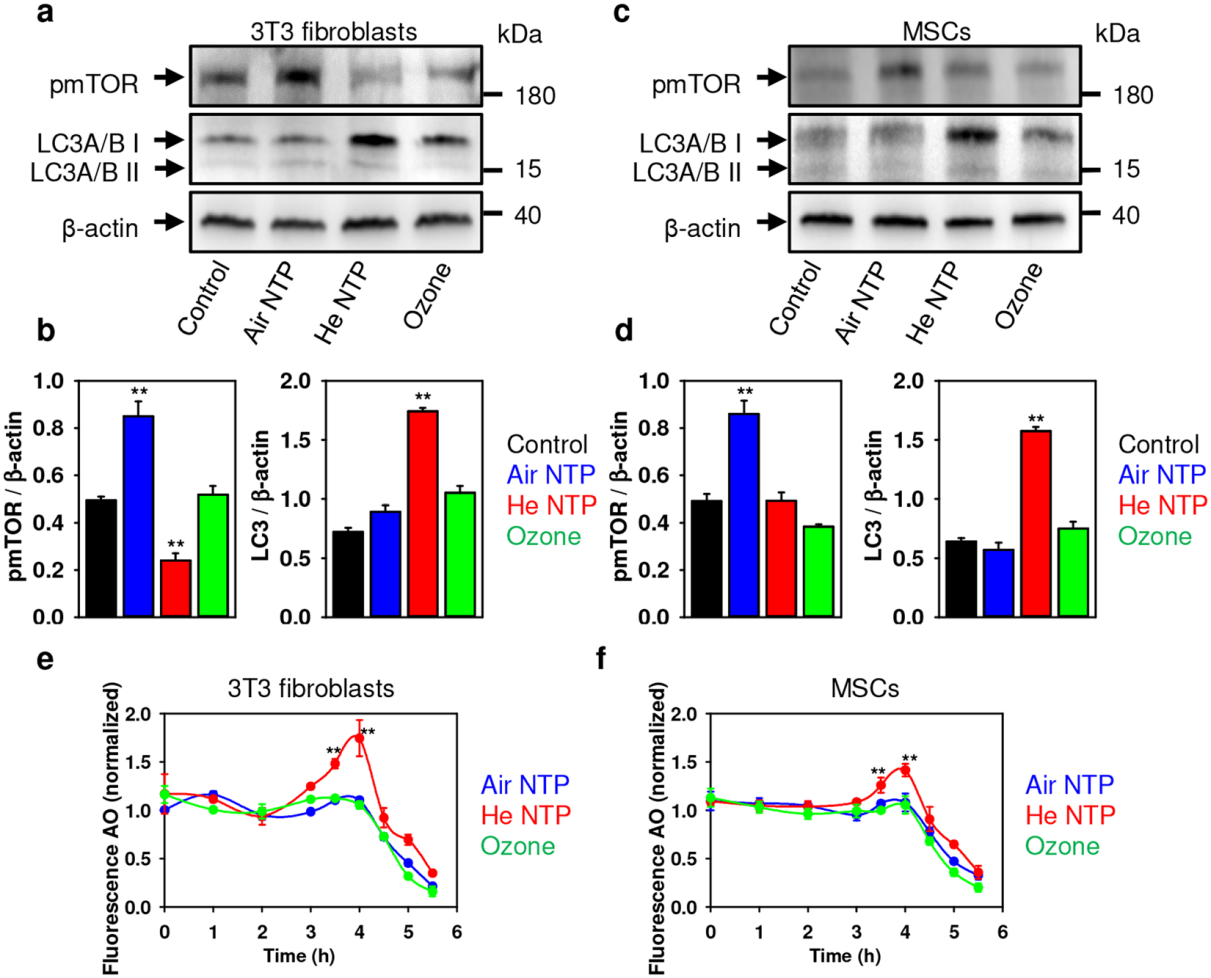
Effects of air, helium NTPs and ozone treatment on mTOR and LC3 activation. (**a**) 3T3 fibroblasts and (**c**) MSCs were treated with air, helium NTPs or ozone for 30 s. Cells were analyzed by Western immunoblotting 4 h after treatment. Actin – control of equal protein loading. Representative blots out of three independent experiments are shown. The graphs show densitometric quantification of the respective Western immunoblots for 3T3 fibroblasts (**b**) and MSCs (**d**), **P < 0.01, mean ± SEM, n = 3. Air, helium NTPs and ozone effects on lysosomal integrity in 3T3 fibroblasts (**e**) and MSCs (**f**). After treatment with NTPs and ozone, cells were stained with acridine orange (AO). AO uptake in acidic lysosomes leads to red fluorescence, which dissipates when the dye leaves this compartment. The accompanying decrease in fluorescence intensity was analyzed by spectrofluorometry. The results are presented as the mean ± SEM of four independent experiments *P < 0.05 **P < 0.01 versus controls.

Importantly, it has been shown that necroptosis could be triggered by promoting the assembly of the necrosome on autophagosomes^52^. Therefore, we studied markers of autophagy under both NTPs and ozone treatments. Indeed, only He NTP treatment resulted in up-regulation of LC3 (an autophagy marker) and formation of autophagosomes (Figs 6a–d and S4 in Supporting Information). Moreover, only He NTP treatment induced lysosomal acidification, a prerequisite of autophagy (Fig. 6e,f). Taken together, these data confirm that He NTP induces necroptotic cell death.

However, we still could not explain cytotoxicity triggered by ozone and air plasma. Thus, we investigated further other potential biochemical targets that could be responsible for observed cytotoxicity. Interestingly, we found the mechanistic target of rapamycin (mTOR) activation upon air NTP treatment without concomitant autophagy activation (Fig. 6a–d). These data led us to the conclusion that air NTP treatment results in mTOR-related necrosis. It has been shown that activation of the mTOR signaling pathway promotes necrotic cell death via suppression of autophagy^53^. Of note, air NTP treatment did not induce lysosomal acidification (a well-known inhibition process of mTOR activity) (Fig. 6e,f). Constellation of the following evidence from different assay, namely: mTOR activation without concomitant LC3 upregulation (Fig. 6a–d), absence of STAT1 activation and MLKL phosphorylation (Fig. 5) and no signs of necroptosis execution (Fig. 4), led us to the reasonable conclusion that air NTP treatment results inmTOR-related necrosis.

**Figure 7:**
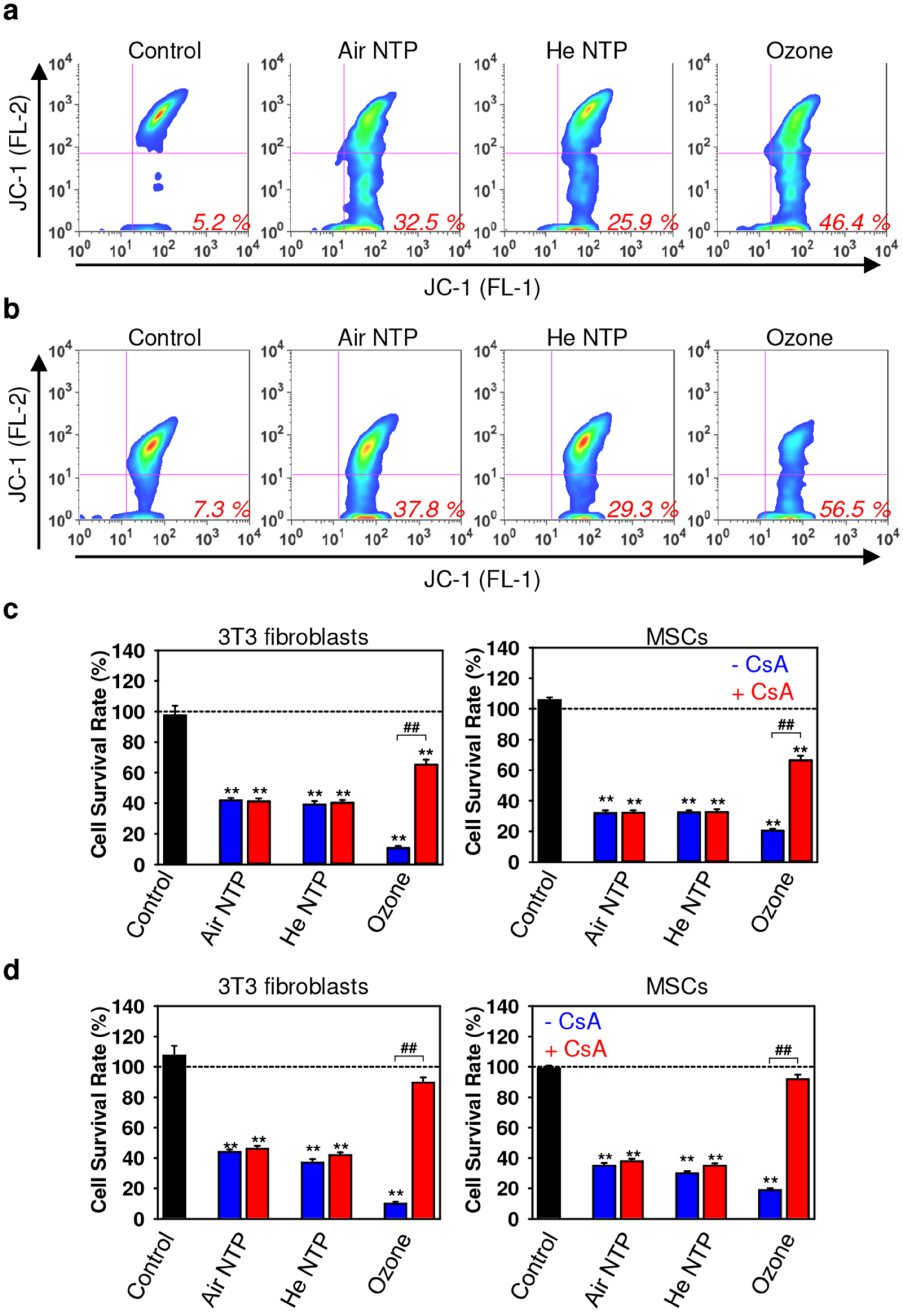
Ozone mediates CypD-related necrosis. Air, helium NTPs and ozone treatments induce mitochondrial dysfunction in 3T3 fibroblasts (**a**) and MSCs (**b**). Cells were stimulated with different NTPs and ozone then stained with 2 μM JC-1 for 30 min, and analyzed by flow cytometry. Numbers show amounts of JC-1 cells with redlow fluorescence exhibiting damaged mitochondria. Representative flow charts out of three independent experiments are shown. (**c**) Cyclosporin A (CsA, an inhibitor of the mitochondrial permeability transition – mPT) antagonizes the ozone-induced cytotoxicity. Cell viability as detected by the WST-1 assay of 3T3 fibroblasts and MSCs treated with air, helium NTPs or ozone for 60 s with supplementation of 10 µM CsA, measured 24 h after exposure. Readings were done in quadruplicates, data are present as mean ± SD, n = 4 (four independent experiments), *P < 0.05 **P < 0.01. (**d**) Appropriate CsA dose completely eliminates ozone-induced cell death. Cell viability as detected by the WST-1 assay of 3T3 fibroblasts and MSCs treated with air, helium NTPs or ozone for 60 s with supplementation of 20 µM CsA, measured 24 h after exposure. Readings were done in quadruplicates, data are present as mean ± SD, n = 4 (four independent experiments), *P < 0.05 **P < 0.01.

Now the question remained; what kind of biochemical pathway is affected by ozone. Results, showing RIP3-RIP1 necrosome formation upon ozone treatment (Fig. 4d) in combination with infectivity of cytotoxicity inhibition by Nec-1 (Fig. 4a,b) and absence of MLKL phosphorylation (Fig. 5c,d), led us to hypothesize that ozone might induce mitochondria related necrosis. Thus, we explored whether NTPs and ozone cause mitochondrial dysfunction. To investigate whether NTPs and ozone can perturb mitochondrial function, we used the fluorescent dye JC-1(a cationic dye that exhibits a potential-dependent accumulation in mitochondria). As expected, both NTPs and ozone induced depolarization of the mitochondrial membrane, as indicated by a decrease of the red-to-green fluorescence intensity ratio (Fig. 7a,b). However, ozone was the most aggressive compound inducing the highest damage (Fig. 7a,b). Apart from mitochondrial depolarization, ozone also induced the highest ROS/RNS levels (Fig. 1c,d), and as we previously showed^5^ – the highest superoxide (O_2_^−^) accumulation. All of these data clearly demonstrate mitochondrial involvement in ozone-triggered cell death. Indeed, ozone-induced cytotoxicity inhibition by specific cyclophilin D (CypD) and pharmacological inhibitor cyclosporin A (CsA), revealed that ozone triggers CypD-related necrosis via the mitochondrial permeability transition (mPT) (Fig. 7c). Indeed, the inhibition of ozone-induced cytotoxicity by CsA was not complete (Fig. 7c). However, pharmacological inhibition efficacy is greatly dependent on the concentration of the used drug^54^. Thus, in order to fully support our hypothesis of CypD-related necrosis, we performed additional cytotoxicity inhibition utilizing a higher dose of CsA (Fig. 7d). Of note, a higher dose of CsA completely eliminated ozone-induced cell death (Fig. 7d). Importantly, there is substantial evidence showing a clear separation of necroptosis from CypD-mediated regulated necrosis^42, 55^. This explains, observed by us, the non-active necrosome complex formation after ozone treatment (Fig. 4c). Of note, necroptosis and CypD-mediated necrosis have a signaling crosstalk between these biochemical cascades^42, 55^.

## Discussion

**Figure 8:**
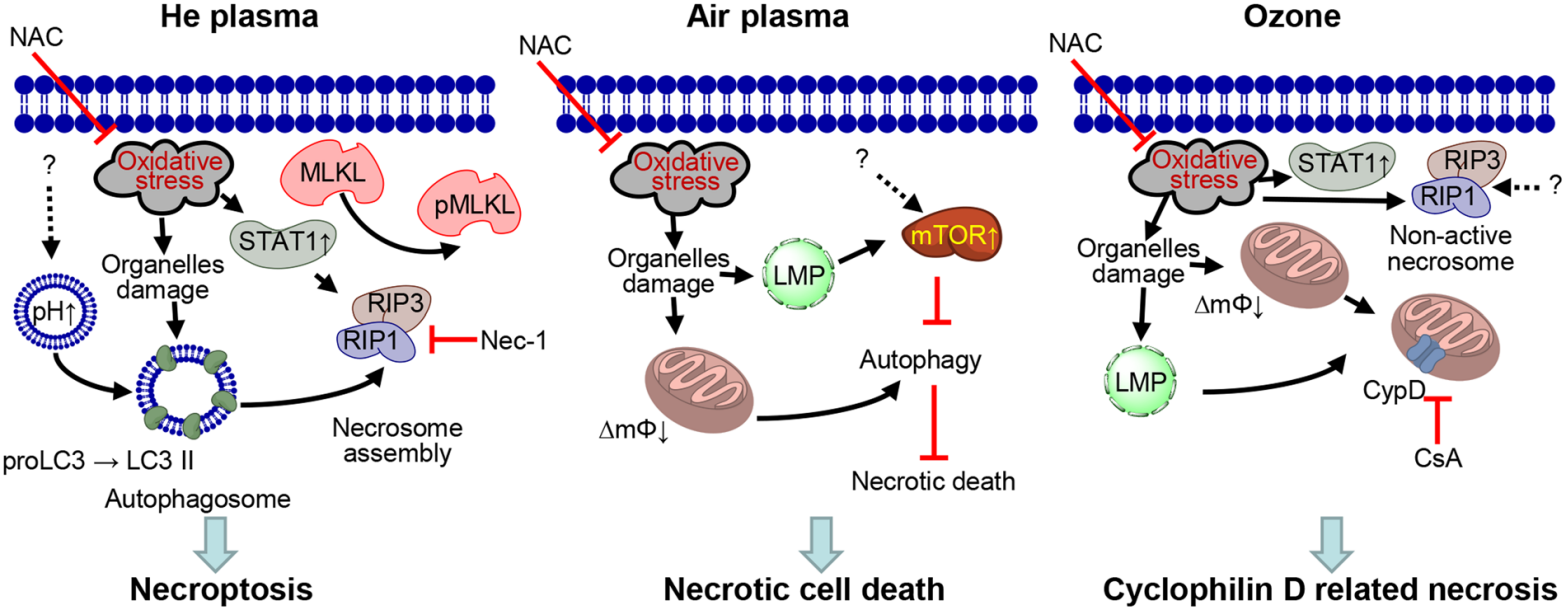
Scheme of district biochemical signaling activation in cells after stimulation with air, helium NTPs and ozone. LMP – lysosomal membrane permeabilization; ∆mΦ – mitochondrial membrane potential; CsA – cyclosporin A; LMP – lysosomal membrane permeabilization.

On the basis of our results, we propose the following biochemical mechanisms of air and He NTPs, and ozone action on living cells (Fig. 8). Helium NTP treatment results in ROS/RNS accumulation in cells which leads to oxidative stress. Such oxidative stress triggers autophagy (the cell is trying to cope with damaged structures) activation that supports necrosome formation and necroptosis execution (Fig. 8). Contrarily, oxidative stress promoted by air NTP leads to mTOR activation and associated with it; autophagy inhibition, that induces mTOR-related necrosis. Importantly, upon air NTP treatment, there was a significant mTOR activation without concomitant LC3 upregulation (Fig. 6a–d) and lysosomal acidification (Fig. 6e,f). Further, air NTP did not result in either STAT1 activation or MLKL phosphorylation (Fig. 5). Additionally, there were no signs of necroptosis execution (Fig. 4) after air NTP treatment. Therefore, we concluded that air NTP treatment results in mTOR-related necrosis.

Interestingly, ozone due to the highest levels of ROS production mediates CypD-related necrosis via the mPT (Fig. 8). However, there was a substantial overlap in biochemical cascades activated by He NTP and ozone. Both treatments resulted in RIP1/RIP3 upregulation and the formation of RIP3-RIP1 complexes (Fig. 4d). He NTP and ozone induced STAT1 phosphorylation (Fig. 5a,b). Indeed, the initiation of necroptosis requires the kinase activity of RIP1 and RIP3, and its execution involves the active disintegration of mitochondrial, lysosomal and plasma membranes^56^. Of note, both He NTP and ozone induced mitochondrial damage (Fig. 7a,b), lysosomal leakage (Fig. 6e,f) and plasma membrane disintegration (Fig. 3a–d), showing substantial crosstalk in cell death pathways. Indeed, inhibition of CypD completely eliminated ozone-induced cell death (Fig. 7d). Contrarily, inhibition of CypD did not result in any increase in cell survival after He NTP treatment (Fig. 7d). On the other hand, recent studies suggest that cyclophilin D might be a downstream target of the necrosome^57^. However, CypD-related necrosis via the mPT and necroptosis are clearly shown to be separate pathways^58^. Despite similarities in activation of cellular processes by He NTP and ozone, these treatments possess crucial differences. First of all, intracellular ROS accumulation is substantially higher after ozone treatment in comparison with He NTP (Fig. 1c,d). Ozone results in significantly higher DNA oxidative damage in contrast to He NTP (Fig. 3b,d). Importantly, after ozone treatment RIP3-RIP1 necrosome complex is not active and does not phosphorylate MLKL (Fig. 5c,d and Fig. S3 in Supporting Information). Moreover, CsA completely abolishes the toxicity induced by ozone, whereas it has no effect on He NTP-treated cells (Fig. 7d). All these functional data allow us to conclude that ozone mediates CypD-related necrosis via the mPT, whereas He NTP executes necroptosis.

It is well-known, that high levels of ROS may induce not only intracellular signals, but can also damage cellular structures, finally leading to distinct types of cell death^41–43, 59^. However, it is difficult to understand how redox signaling achieves specificity upon distinct NTPs and ozone treatment, taking into account the chemical simplicity of most ROS. A recent study shows that oxidants and their targets might be spatially confined within the cell^60^. Moreover, we have previously shown that air and He NTPs possess different ion penetration profiles^5, 21^. In addition, the ROS/RNS composition and concentrations range in the plasma-treated liquids are variable, depending on the carrier gas that forms NTP^9, 61^. For instance we showed previously that air NTP bears higher levels of nitric oxide (NO) in comparison with He NTP^5, 21^. Of note, NO has been shown to drive mTOR pathway activation^62^. Thus, it is no surprise that air, He NTPs and ozone trigger distinct biochemical pathways of cell death. Importantly, these types of cell death are morphologically indistinguishable, and only rigorous analysis of their biochemical marker can decouple them^18, 42, 43^.

## Conclusion

In summary, we have demonstrated that cell exposure to NTPs or ozone led to activation of distinct biochemical signaling, that results in triggering the specific cell death pathways. Generally, different cell death scenarios could be initiated by various external physical cues such as temperature, ultraviolet light^63^, electric^64^ and magnetic fields^65^. Among these cues, non-thermal plasmas hold benefits for biomedical applications due to a variety of adjustable physical parameters: chemical composition, power, ion density, torch geometry, gas temperature, ROS concentration, etc. Playing with these parameters, one can achieve activation of specific biochemical pathways in living cells. As shown here, by changing the plasma composition we triggered two distinct cell death pathways. Despite similar ROS level production, air and He NTPs induce either mTOR-related necrosis or necroptosis. Our results shed light on the identification of molecular targets upon NTP treatment of living cells. We have also demonstrated that the NTP-mediated cell toxicity can be abolished by the therapeutic ROS scavenger NAC. Hence, these results imply that the cytotoxic effects of NTPs require more intensive study, which should be considered when such plasmas are intended for use in biomedical applications.

## Materials and Methods

### Physicochemical characterization of the plasma

To produce uniform non-thermal plasma for biological applications, we utilized the plasma setup published in refs 5 and 21. The input voltage was about 600 V, electric current 167 mA, and the power was 100 W; such a high voltage supply resulted in electron energy of about 0.5 keV. The gas supply was administered through a gas inlet followed by gas ionization in the pores of the ceramic membrane, utilizing an electric field between two electrodes. The total helium or air flow through the nozzle was set to 4 L min^−1^ for each gas. The same flow rate was for the ozone generator. The emitting plasmas were well spatially localized, showing the applicability of the proposed plasma reactor for the controlled treatments of cells and tissue. The gas temperature of such plasma jets was measured by a K type thermocouple at a 10 mm distance from the nozzle, and the temperature did not exceed 36 °C during the entire treatment time. Thus, a limited gas temperature implies the absence of thermal damage on living cells or tissue during plasma treatments. FTIR experiments were carried out using a Nicolet™ iS™5 FT-IR spectrometer (Thermo Scientific). To compare the effects of plasma with ozone, cells were exposed to ozone produced by an ozone generator with input voltage 230 V, power 12 W and ozone gas production rate 400 mg h^−1^ (FORTEZON 400, UVC servis). Ozone concentration was measured using a gas detector (GasAlert Extreme, BW Technologies). Detailed characteristics of the system have been published previously^5, 20, 21^.

### Cell culture

3T3 fibroblasts (American Type Culture Collection) were grown in culture medium containing Dulbecco’s modified Eagle’s medium (DMEM; PAA Laboratories, Pasching, Austria), with 10% fetal bovine serum (FBS; PAA Laboratories) and Primocin TM (100 µg/ml; Lonza, Cologne, Germany). Mesenchymal stem cells (MSCs) were isolated from human umbilical cords obtained from healthy full-term neonates after spontaneous delivery with the informed consent of the donors, using the guidelines approved by the Institutional Ethics Committee at University Hospitals (Pilsen and Prague, Czech Republic). All methods were performed in accordance with the relevant guidelines and regulations of the Institutional Ethics Committee at University Hospitals (Pilsen and Prague, Czech Republic). MSCs were isolated according to previously published protocol^66^ and expanded in alpha-MEM (EastPort, Prague, Czech Republic), supplemented with 5% platelet lysate (IKEM, Prague, Czech Republic) and gentamicine 10 µg/ml (Sandoz, Prague, Czech Republic). Cells were cultivated at 37 °C in a humidified atmosphere containing 5% CO_2_, and the medium was changed twice a week.

### Plasma treatment

Non-thermal plasma was produced using a system with a nozzle array to treat living cells as illustrated by Figure S1. A generalized sketch is shown in Figure S1a, and a detailed plasma treatment procedure is depicted in Figure S1b,c. In order to alter the chemical composition of the plasma, we used either an air or helium gas supply. Gas was supplied through a gas inlet and then was ionized in the pores of the ceramic membrane. Cells grown to 70% confluence were exposed to plasma from the device located 10 mm away, for 15, 30 and 60 s. Before the treatment, the medium was removed from culture wells and then 50 µl of medium was added to prevent the cells from drying up. After the plasma treatment, the remaining medium was replaced with a fresh one. The majority of experiments were done in 96-well plates in quadruplicates, with at least 3 or 4 independent experiments. Immunofluorescence and immunoblot assay were done on cells irradiated by plasma in 24-well plates. In order to avoid cross-stimulation of neighboring wells, we treated wells by plasma through a protective plastic sheet (2 mm thickness) with a hole of the same size as the plasma nozzle (see Fig. S1b in Supporting Information). Moreover, we seeded cells with one well gap filled with sterile water to be sure that different exposures were not cross-reacting (see Fig. S1c in Supporting Information).

Further, to make plasmas spatially well localized, we developed a plastic concentrator for the nozzle (see Fig. S1b in Supporting Information). Being aware that NTPs might change the pH of liquids, we monitored pH changes upon plasma treatment. The pH of the media solution was measured with a pH probe (Oakton pHTestr30) before and after the solution was treated with the plasma, for a given amount of time (15, 30 and 60 s). All irradiation times by both plasmas did not change the pH of the cell culture media; the pH was at the range of 7.4. This observation is not surprising, because it has been shown that short-term exposure for less than 4 min does not lead to pH changes in media^9, 23, 24^. Additionally, we performed power measurements of UV production by air and helium NTPs utilizing UV light meter (Lutron YK 35UV). Power measurements of UV production, where the power density for air was lower than 1 μW/cm^2^ and for He NTP– 3 ± 1 μW/cm^2^, which is at least one order of magnitude lower then the minimal power density needed to have any effect on living cells^25–27^.

### Measurement of cellular viability

Cell viability was analyzed by WST-1 assay (Roche Diagnostics), which is based on the cleavage of tetrazolium salt WST-1 by cellular mitochondrial dehydrogenases, producing a soluble formazan salt; this conversion only occurs in viable cells, thus allowing an accurate spectrophotometric quantification of the number of metabolically active cells in the culture. Cells were seeded onto 96-well plates at a density of 8000 cells per well and treated with plasma or ozone. 24 h after the treatment, WST-1 reagent was added to each dish and incubated for 2 h at 37 °C to form formazan. The absorbance was measured using a Tecan-Spectra ELISA plate reader (Mannedorf, Switzerland) at 450 nm. Readings were done in quadruplicates; three independent experiments were performed for each measurement. In order to block ROS/RNS, the ROS scavenging agent 5 mM *N-acetyl-L-cysteine* (NAC) was added to the complete culture medium. In experiments with pharmacological inhibitors, culture medium was supplemented with either 10 µM necrostatin-1 (Nec-1, a potent and selective inhibitor of necroptosis), 10 or 20 µM cyclosporin A (CsA, an inhibitor of the mitochondrial permeability transition – mPT).

### Detection of intracellular ROS and RNS

ROS and RNS levels were measured using Cellular ROS/Superoxide Detection Assay Kit (Abcam). Briefly, cells were seeded onto 96-well black/clear bottom plates (Corning, BD Biosciences) at a density of 8000 cells per well. Following this, plasma treatment cells were labeled with Oxidative Stress Detection Reagent (Green) for ROS detection and Superoxide Detection Reagent (Orange) according to the manufacturer’s instructions (Abcam). Fluorescence was then measured using a fluorescent microplate reader (Tecan Infinite® 200 PRO). Readings were done in quadruplicates. Quantification of ROS levels was done using the methods published earlier^40^.

### Apoptosis assay

Apoptosis was assessed via annexin V/propidium iodide staining. Cells were treated with different plasmas and ozone for 60 s and incubated further for 4 h. Phosphatidylserine expression, as an early sign of apoptosis, was determined via fluorescence microscopy analysis by the binding of fluorescein isothiocyanate-labeled annexin V (Sigma-Aldrich); propidium iodide was used to differentiate necrotic cells. Hoechst was used as nuclear staining. Fluorescence images were recorded with a Zeiss Axioscope 2 microscope (Carl Zeiss AG). ImageJ software (NIH, Bethesda, MD, USA) was used for image processing and fluorescent micrograph quantification. PI and annexin V fluorescence were calculated by normalizing the corrected total cell fluorescence (CTCF) of the full area of interest to average fluorescence of the region. The net average CTCF intensity of a pixel in the region of interest was calculated for each image utilizing a previously described method^67^. The region placed in an area without fluorescent objects was used for background subtraction. CTCF was determined as the sum of pixel intensity for a single image, with the subtracted average signal per pixel for a region selected as the background.

### Caspase-3 activity assay

As an apoptosis parameter, caspase-3 activation was detected; the caspase-3 inhibitor VAD-FMK conjugated to FITC (FITC-VAD-FMK) as a marker. FITC-VAD-FMK is cell permeable, nontoxic, and irreversibly binds to activated caspases in apoptotic cells. After 4 h post NTPs and ozone treatment, cells were loaded with FITC-conjugated, pan-caspase inhibitor peptide, FITC-VAD-FMK (Abcam), according to the manufacturer’s instructions. Following staining, cells were analyzed using a fluorescent microplate reader (Tecan Infinite® 200 PRO). Readings were done in quadruplicates. As a positive control, cells were treated with 2 μM staurosporine for 3 h.

### Quantification of mitochondrial membrane potential

Plasma-treated cells were further incubated for 4 h to measure mitochondrial membrane potential (ΔmΦ). After 4 h of incubation, cells were loaded with 2 µM JC-1 (Invitrogen), a lipophilic cationic fluorescence dye with a dual emission wavelength for 30 min, in order to analyze the depolarization of the ΔmΦ. At low concentrations (due to low ΔmΦ), JC-1 is predominantly a monomer that results in a green fluorescence with an emission of 530 nm. At high concentrations (due to high ΔmΦ) the dye aggregates, yielding a red emission of 590 nm. Thus a decrease in the aggregate fluorescent count displays a mitochondrial membrane depolarization, whereas an increase exhibits a hyperpolarization. Following staining, cells were analyzed using a flow cytometry using an Apogee Flow Cytometer (Auto 40).

### Assessment of lysosomal integrity by Acridine Orange (AO) release

Cells were labeled with 5 µg/ml AO in DMEM culture medium for 15 min at 37 °C. After the rinsing of cells in complete medium, they were exposed to different types of plasma and ozone. Following this, cells were cultured at 37 °C for indicated periods of time, and then orange fluorescence intensity was measured using a fluorescent microplate reader (Tecan Infinite® 200 PRO). Readings were done in quadruplicates.

### Cell extracts and western immunoblot analysis

Aliquots of whole cell lysates^68^ containing equal amounts of protein were separated by SDS-PAGE, transferred, probed with specific antibodies against RIP1 (Biorbyt Ltd.), RIP3 and caspase-8 (both from Abcam), phospho-STAT1 and phospho-mTOR (both from Cell Signaling Technologies), STAT1 (Santa Cruz), caspase-3, LC3A/B and phosphor-MLKL (all from Cell Signaling Technologies) and detected as described^69^. Actin (Thermo Fisher Scientific) staining served as a loading control.

### Immunofluorescence

For immunostaining and epi-fluorescent microscopy, cells after ait, helium NTPs or ozone exp were fixed with 2% formaldehyde and permeabilized with 0.2% Triton X-100. Subsequently, cells were stained either with rabbit polyclonal anti-phospho-MLKL (polyclonal, clone D6H3V, Cell Signaling Technologies), or rabbit polyclonal LC3A/B antibody (polyclonal, Cell Signaling Technologies), with subsequent labelling using secondary Alexa Fluor-568 antibodies (Invitrogen). Nuclei were counterstained with Hoechst 33342. Samples were covered with mounting medium (Dako) and analyzed by epi-fluorescent microscopy (Eclipse Ni-E, Nikon, Japan). ImageJ software was used for image processing and quantification.

### Co-immunoprecipitation

After 4 h post NTPs and ozone treatment, cells were lysed with lysis buffer from immunoprecipitationkit (Abcam). RIP3-RIP1 complexes were co-immunoprecipitated from the precleared cell lysates with the appropriate Ab as described in the manufacturer’s instructions. After pre-clearing with Protein A/G Sepharose^®^ beads, the lysates were immunoprecipitated with anti-RIP3 antibody for 12 hr and washed. The resulting protein complex was eluted from the beads with Laemmli protein sample buffer for SDS-PAGE (Bio-Rad) and resolved on SDS-PAGE.

### Immunocytochemistry DNA damage

Cells were cultured in a 24 well plate on glass cover slips coated with laminin (0.1% gelatine), treated with different plasmas and ozone for 30 s and incubated for 1, 4 and 24 h. Cell were then fixed in 4% paraformaldehyde in 0.1 M PBS for 15 min and washed with 0.1 M PBS. Non-specific staining was avoided by application of blocking goat serum (1:10; G9023, Sigma Aldrich, St. Louis, MO, USA) and Triton X-100 (0.1%; T8787, Sigma Aldrich, St. Louis, MO, USA) in 0.1 M PBS (45 min., RT). Cells were treated with anti-phospho-histone H2A.X mouse monoclonal antibody (1:500; MILL05-636, Merck Millipore, Darmstadt, Germany), diluted in 0.1 M PBS containing goat serum (1:10) and Triton X-100 (0.1%) for 15 min in RT followed by 75 min incubation in 4 °C. After washing with Triton X-100 (0.1%) in 0.1 M PBS, cells were incubated (2 h, 4 °C, in dark) with secondary goat anti-mouse IgG antibody conjugated with Alexa-Fluor 488 (1:400; A11029, Life Technologies, Eugene, OR, USA), diluted in 0.1 M PBS containing goat serum (1:10) and Triton X-100 (0.1%). Additional fluorescent nuclear staining was accomplished with DAPI (1:1000; D1306, Life Technologies, Eugene, OR, USA) and Triton X-100 (0.1%) diluted in 0.1 M PBS. The culture slides with stained cells were mounted with Aqua Poly/Mount (18606, Polysciences, Warrington, PA, USA). Fluorescent micrographs were taken using an LSM 510 DUO laser scanning confocal microscope (Zeiss).

For quantitative analysis, fluorescence images were recorded with an AxioCam HRc Axioskop 2 Plus fluorescence microscope (Zeiss, Jena, Germany) using a 20x objective. Three images from each sample were taken. The experiment was done in duplicates. ImageJ software was used for image processing and fluorescent micrograph quantification. Quantitative analysis was carried out by counting the number of immunoreactive cells as the percentage of the total number of viable cells as determined by DAPI staining.

### Statistical analysis

Quantitative results are expressed as mean ± SEM. Results were analyzed by multi-group comparison Fisher’s LSD and Newman-Keuls tests. Differences were considered statistically significant at *P < 0.05.

## Electronic supplementary material

Supplementary Information
