# Supplementary material for: Chemically different non-thermal plasmas target distinct cell death pathways
Source: Sci Rep. 2017 Apr 4;7:600. doi: 10.1038/s41598-017-00689-5 (PMC5428849; doi:10.1038/s41598-017-00689-5)
Supplement: Supplementary file 1 — Supplementary Information [file 41598_2017_689_MOESM1_ESM.pdf]

Supporting Information Online

Chemically different non-thermal plasmas target distinct cell death pathways

Oleg Lunov[1], Vitalii Zablotskii[1], Olexander Churpita[1], Mariia Lunova[2], Milan Jirsa[2], Alexandr Dejneka[1] and Šárka Kubinová[1,3]

[1]Institute of Physics of the Academy of Sciences of the Czech Republic, Prague, 18221, Czech Republic.
[2]Institute for Clinical & Experimental Medicine (IKEM), Prague, 14021, Czech Republic.
[3]Institute of Experimental Medicine AS CR, Prague, 14220, Czech Republic.

Corresponding author:

Correspondence and requests for materials should be addressed to O.L. (email: lunov@fzu.cz).

Institute of Physics of the Academy of Sciences of the Czech Republic, Prague, 18221, Czech Republic.

Tel: +420266052131

E-mail: lunov@fzu.cz

Supplementary Figures

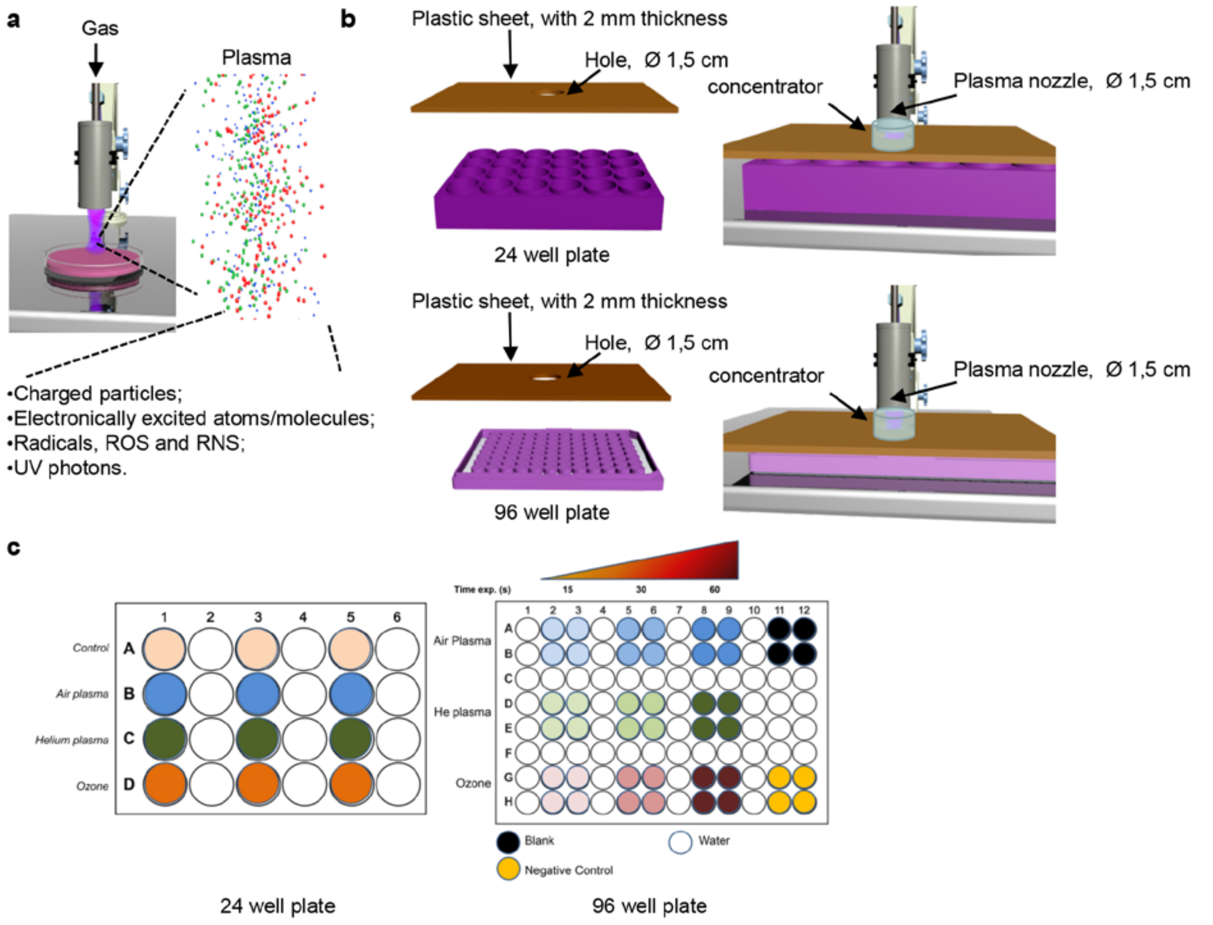

Figure S1 Scheme of the plasma device and experimental setup. (a) Generalized sketch of non-thermal plasma application. (b) Schematic diagram of plasma action on cell culture. (c) Representative culture plate schemes of non-thermal plasma application.

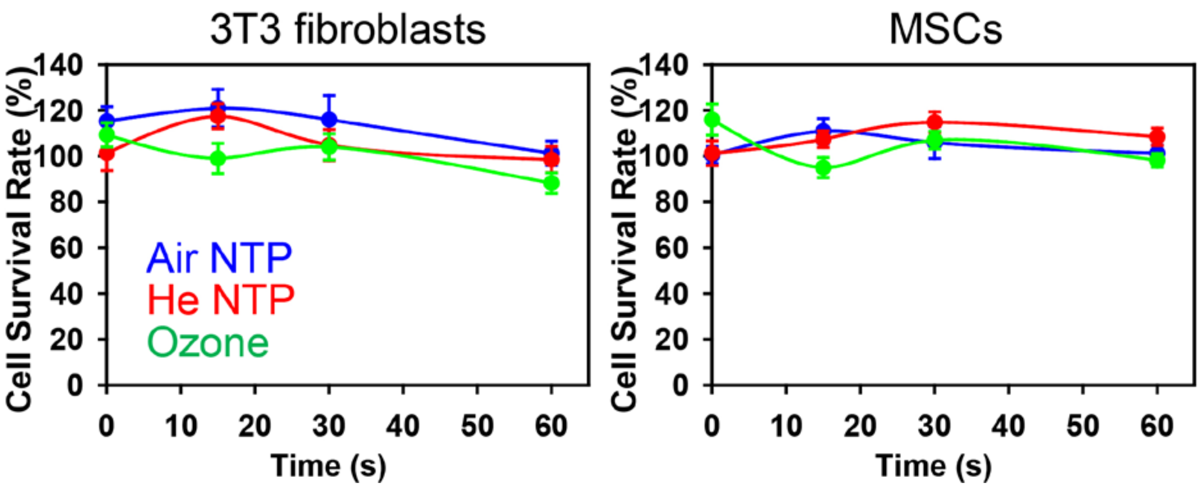

Figure S2 ROS scavenger (*N-acetyl-L-cysteine* NAC) reduces the cytotoxicity induced by air, helium NTPs and ozone. Cell viability as detected by the WST-1 assay of 3T3 fibroblasts and MSCs treated with air, helium NTPs or ozone for indicated time periods with supplementation of 5 mM NAC, measured 24 h after exposure.

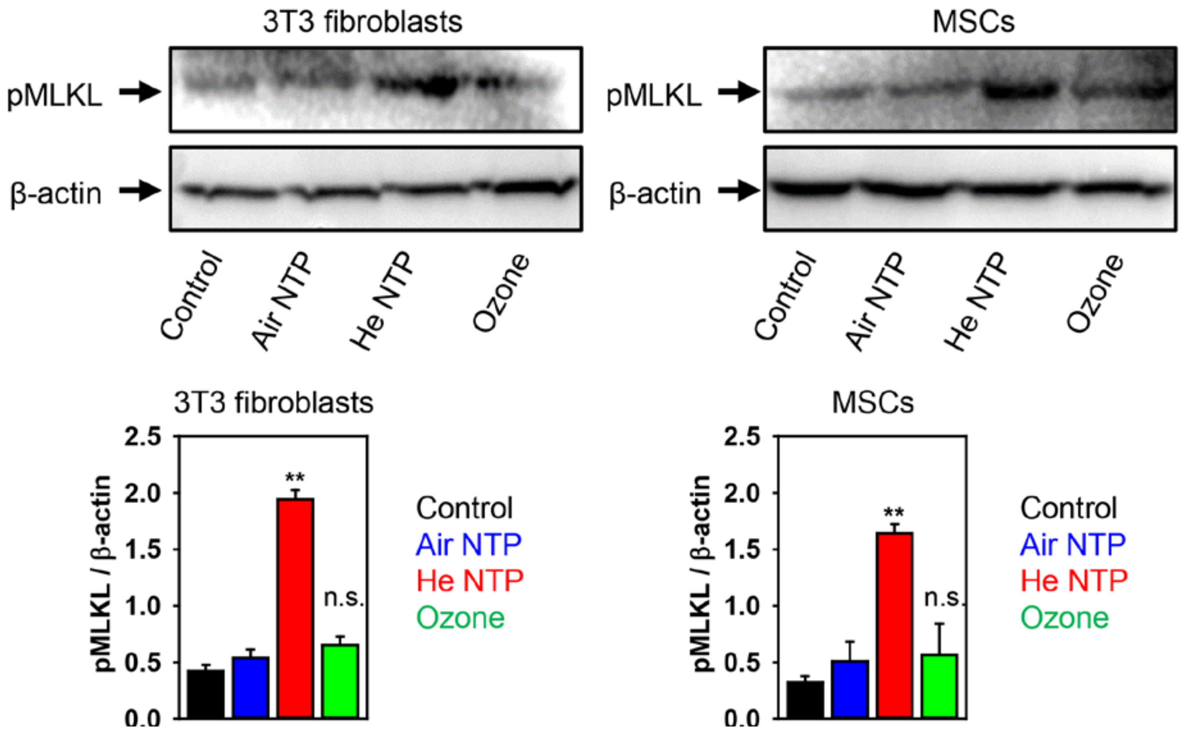

Figure S3 Effects of air, helium NTPs and ozone on MLKL activation. 3T3 fibroblasts and MSCs were treated with air, helium NTPs or ozone for 30 s. Cells were analyzed by Western immunoblotting 4 h after treatment. Actin – control of equal protein loading. The graph shows densitometric quantification of the respective immunoblots, *$P < 0.05$ **$P < 0.01$, mean ± SEM, n=3. Representative blots out of three independent experiments are shown.

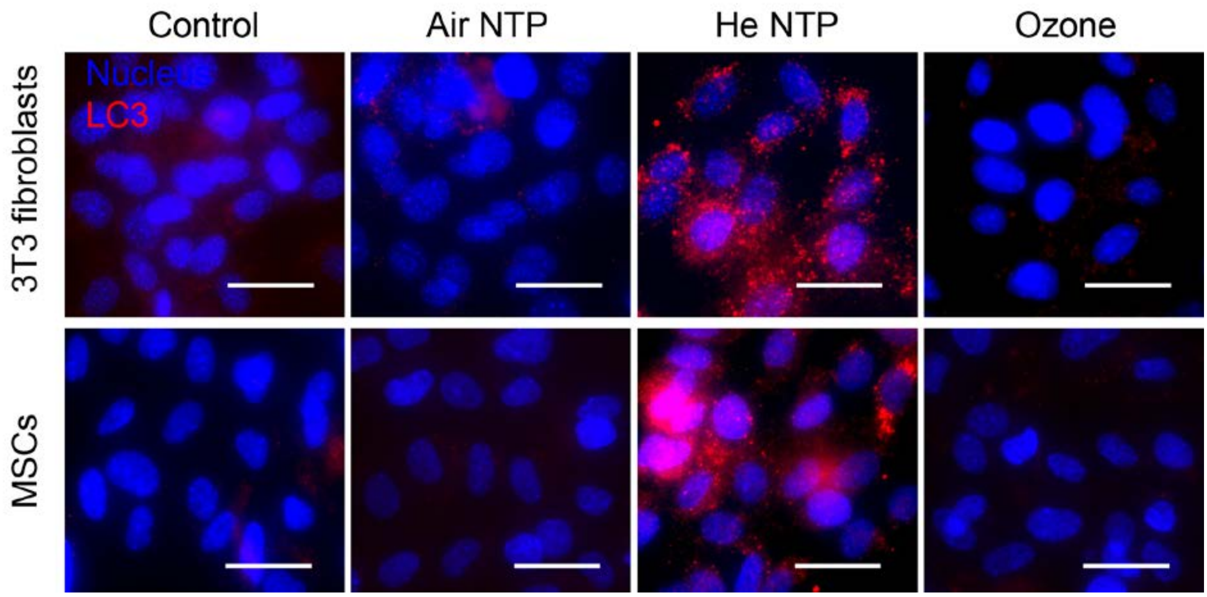

Figure S4 Effects of air, helium NTPs and ozone on autophagy. Representative pictures of 3T3 fibroblasts and MSCs treated with air, helium NTPs or ozone for 30 s. Cell were stained for nuclei (blue), and LC3 (red). Labeled cells were then imaged using epi-fluorescent microscopy, and the image was processed with ImageJ software (NIH, Bethesda, MD, USA). Scale bar 50 µm.

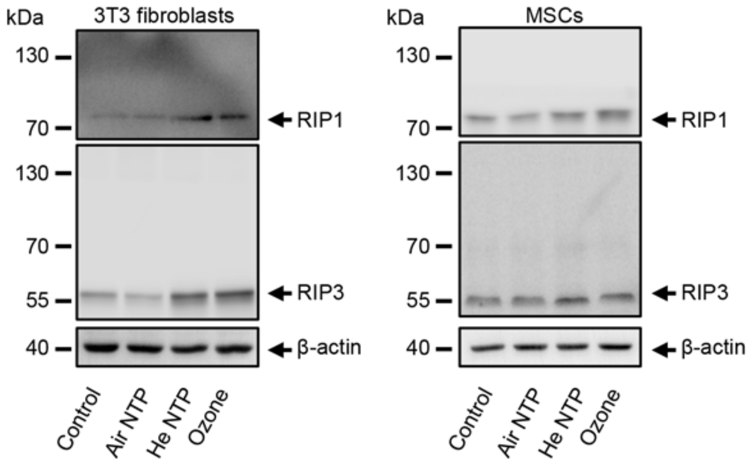

Figure S5 He NTP and ozone treatment induces RIP1 and RIP3 upregulation. 3T3 fibroblasts and MSCs were treated with air, helium NTPs or ozone for 30 s. Cells were analyzed by Western immunoblotting 4 h after treatment. Actin – control of equal protein loading. Representative blots out of three independent experiments are shown.
